# Ethanol Cellular Defense Induce Unfolded Protein Response in Yeast

**DOI:** 10.3389/fmicb.2016.00189

**Published:** 2016-02-18

**Authors:** Elisabet Navarro-Tapia, Rebeca K. Nana, Amparo Querol, Roberto Pérez-Torrado

**Affiliations:** Instituto de Agroquímica y Tecnología de los Alimentos-Consejo Superior de Investigaciones CientíficasValencia, Spain

**Keywords:** yeasts, *Saccharomyces*, ethanol stress, transcriptomics, UPR

## Abstract

Ethanol is a valuable industrial product and a common metabolite used by many cell types. However, this molecule produces high levels of cytotoxicity affecting cellular performance at several levels. In the presence of ethanol, cells must adjust some of their components, such as the membrane lipids to maintain homeostasis. In the case of microorganism as *Saccharomyces cerevisiae*, ethanol is one of the principal products of their metabolism and is the main stress factor during fermentation. Although, many efforts have been made, mechanisms of ethanol tolerance are not fully understood and very little evidence is available to date for specific signaling by ethanol in the cell. This work studied two *S. cerevisiae* strains, CECT10094, and Temohaya-MI26, isolated from flor wine and agave fermentation (a traditional fermentation from Mexico) respectively, which differ in ethanol tolerance, in order to understand the molecular mechanisms underlying the ethanol stress response and the reasons for different ethanol tolerance. The transcriptome was analyzed after ethanol stress and, among others, an increased activation of genes related with the unfolded protein response (UPR) and its transcription factor, Hac1p, was observed in the tolerant strain CECT10094. We observed that this strain also resist more UPR agents than Temohaya-MI26 and the UPR-ethanol stress correlation was corroborated observing growth of 15 more strains and discarding UPR correlation with other stresses as thermal or oxidative stress. Furthermore, higher activation of UPR pathway in the tolerant strain CECT10094 was observed using a UPR mCherry reporter. Finally, we observed UPR activation in response to ethanol stress in other *S. cerevisiae* ethanol tolerant strains as the wine strains T73 and EC1118. This work demonstrates that the UPR pathway is activated under ethanol stress occurring in a standard fermentation and links this response to an enhanced ethanol tolerance. Thus, our data suggest that there is a room for ethanol tolerance improvement by enhancing UPR response.

## Introduction

All living organisms are subjected to changing environmental conditions, such as temperature, humidity, and salinity, which can affect optimal growth and reproduction conditions. Cells have developed diverse strategies to combat the harmful effects of a variety of stress conditions that depend on a complex network of sensors and signal transduction pathways, which lead to adaptation in cell cycle, and also to adjustments in gene expression profiles and cell metabolic activities. Although, *Saccharomyces cerevisiae* is a traditional ethanol-producing microbe widely used for the production of bioethanol, alcoholic beverages, and other industrial products, this yeast is also sensible to ethanol that negatively influences the fermentation kinetics (Ansanay-Galeote et al., 2001). Ethanol is a two-carbon alcohol which, due to its small size and alcoholic hydroxyl group, is soluble in both aqueous and lipid environments, which allows it to pass into cells through the plasma membrane increasing fluidity and permeability. Ethanol disturbance of cellular membranes involves a number of consequences that affect many cellular functions (Brooks, 2000; Albano, 2006). Although ethanol forms part of metabolism in many organisms, it alters mitochondrial structure, lowers respiratory rates and ATP levels, and elicits the formation of reactive oxygen species (ROS) and acetaldehyde, which ultimately generate DNA damage, lipid peroxidation and oxidative stress, and reduce cell viability (Brooks, 2000; Hoek and Pastorino, 2002; Lamarche et al., 2003; Albano, 2006; Pandol et al., 2010). Several studies have provided us with some leads to the molecular basis underlying yeast response and resistance to ethanol stress. A correlation between ethanol resistance and trehalose, proline and ergosterol accumulation to enhance the stability of proteins and membranes, and the influence of the degree of fatty acid unsaturation of membrane lipids to antagonize fluidity by ethanol, have been documented (Alexandre et al., 1994a,b; You et al., 2003). Cell wall remodeling, tryptophan biosynthesis, and induction of multiple chaperones and heat shock proteins by the oxidative stress and up-regulation of the genes related with NADH/NADPH regeneration to assure a good redox balance have also been reported (Rosa and Sá-Correia, 1991; Alexandre et al., 2001; Hirasawa et al., 2007; Yoshikawa et al., 2009; Li et al., 2010; Stanley et al., 2010a). Ethanol also causes intracellular acidification due to the influx of protons through the damaged cell membrane, which triggers the transport of intracellular H^+^ into vacuoles by V-ATPase to maintain intracellular pH homeostasis (Rosa and Sá-Correia, 1991; Rosa and Sa-Correia, 1996; Forgac, 1998). Although many efforts have been made, mechanisms of ethanol tolerance activation are not fully understood and very little evidence is available to date for specific signaling by ethanol in the cell. Transcription factor Msn2p and its homologous Msn4p are involved in the ethanol response in *S. cerevisiae* via a stress response element (STRE), although other stresses, like heat, osmotic shock or oxidative stress, activate this general stress response. Transcription factors Yap1p and Hsf1p, required for oxidative stress tolerance and heat shock response respectively, are also related to ethanol stress given its pleiotropic effects, which shows that many genes up-regulated by ethanol challenges share the transcription binding motifs of Msn2p/Msn4p, Yap1, Hsf1, and Pdr1p/Pdr3p in their upstream sequence (Ma and Liu, 2010a). Takemura et al. (2004) observed that the nuclear localization of the DEAD box protein Rat8p caused by ethanol stress may contribute to the selective export of mRNA in ethanol-stressed cells.

Over the years, a number of approaches have been used to improve alcohol tolerance to elicit certain cellular phenotypes, such as transposon mutagenesis, gene deletions, and gene transcription reprogramming (Takahashi et al., 2001; Kubota et al., 2004; Alper et al., 2006). More recently, global gene expression studies have provided a better understanding of the molecular basis underlying yeast response and resistance to ethanol stress (Alexandre et al., 2001; Chandler et al., 2004; Li et al., 2010; Stanley et al., 2010a). Although genome-wide approaches to reveal ethanol tolerance candidate genes in industrial strains have been adopted (Rossignol et al., 2003; Marks et al., 2008) most studies have focused on laboratory strains with moderate ethanol tolerance and have left aside the physiological diversity of natural or fermentation isolates (Takahashi et al., 2001; Fujita et al., 2006; Teixeira et al., 2009; Stanley et al., 2010a). Furthermore, most of these studies have focused on the response to a short-term ethanol stimulus. However, ethanol stress is a long-term stress with a highly dynamic transcriptional response. Thus, it is essential to study not only early, but also late responses, as there is generally little overlap between the genes transcriptionally induced under stress and those that appear essential for adaptation. In previous studies, we determined significant differences in ethanol tolerance between natural and fermentative *S. cerevisiae* strains, including strains isolated from flor wine and traditional fermentations of Latin America (Arroyo-López et al., 2010).

Considering these data, this work focused on exploiting the physiological characteristics of two fermentative strains, CECT10094 and Temohaya-MI26, isolated from flor wine and agave fermentations, which differ in ethanol tolerance, to understand the molecular mechanisms underlying the ethanol stress response and the reasons for different ethanol tolerance among *S. cerevisiae* strains.

## Methods

### Yeast growth media

The basal growth media selected for the experiments were standard GPY medium (5 g/L yeast extract, 5 g/L peptone, 20 g/L glucose). Media were modified whenever necessary with geneticine (200 μg/ml), tunycamicine (1 μg/ml), H_2_O_2_ (3 mM), beta-mercaptoethanol (0–45 mM), or ethanol [0 and 10% (v/v)].

### Strains and plasmid construction

The strains used in this work are listed in Table 1. The *S. cerevisiae* strains used in the genome-wide analysis, CECT10094 and Temohaya-MI26, belong to our collection and were isolated from different traditional fermentation environments (Santa María, 1968; Arroyo-López et al., 2010). In the previous work, Arroyo-López et al. (2010) had a misidentification and described CECT10094 as PE35M. The 15 *S. cerevisiae* strains used in the correlation study among the different stressors belonged to our own collection. The mCherry UPR reporter with geneticine selective marker was obtained by a marker swap of the commercial plasmid pPM47 (Addgene). KanMX cassette with *URA3* extremes was amplified from pFA6a-KanMX6 plasmid (Addgene) and cotransformed with *Eco*RV digested pRM47 following described procedures (Cross, 1997). Plasmids were recovered and confirmed by PCR and enzyme restriction analysis.

**Table 1:** **Strains used in this work**.

**Strain**	**Species**	**Origin**	**Source**
CECT10094	*S. cerevisiae*	Flor wine (Spain)	Santa María, 1968
Temohaya-MI26	*S. cerevisiae*	Agave fermentation (Mexico)	Arroyo-López et al., 2010
CHR9	*S. cerevisiae*	Forest soil (Hungary)	Arroyo-López et al., 2010
CECT 1942	*S. cerevisiae*	Ale beer (Netherlands)	Belloch et al., 2008
CHR96.2	*S. cerevisiae*	Oak tree bark (Spain)	Arroyo-López et al., 2010
RVA^C^	*S. cerevisiae*	Wine fermentation (Spain)	Arroyo-López et al., 2010
CPE7	*S. cerevisiae*	Sugarcane fermentation (Brazil)	Arroyo-López et al., 2010
CECT 11001	*S. cerevisiae*	Lager beer (Belgium)	Oliveira et al., 2014
Lalvin T73^C^	*S. cerevisiae*	Wine fermentation (Spain)	Oliveira et al., 2014
GB Flor-C	*S. cerevisiae*	Wine fermentation (Spain)	Arroyo-López et al., 2010
EC1118^C^	*S. cerevisiae*	Champagne (France)	Arroyo-López et al., 2010
D14	*S. cerevisiae*	Food supplement	Llopis et al., 2012
60	*S. cerevisiae*	Clinical isolate (Spain)	Llopis et al., 2012
W303	*S. cerevisiae*	Laboratory strain	Our collection
BY4743	*S. cerevisiae*	Laboratory strain	Euroscarf

C *Commercial strain*.

### Growth conditions and experimental design for transcriptome analysis

A single colony of each strain, CECT10094 and Temohaya-MI26, was picked up from fresh GPY-agar plates and separately incubated overnight into 12 mL sterile tubes with 5 mL GPY in an orbital shaker at 150 rpm and 28°C. These cultures in the exponential phase were used to inoculate 100 mL of GPY to an initial OD_600_ of 0.1 into sterile 250 mL Erlenmeyer flasks with cotton wool plugs in the absence or presence of ethanol 10% (v/v), a concentration that significantly affects both strains but allow them to grow (Arroyo-López et al., 2010). The samples for RNA extraction were taken after 1 h (early response) and after exponential phase (late response) under both ethanol-stressed and non-stressed conditions. Samples were taken by centrifugation at 3500 rpm for 2 min, washed with water and frozen in liquid N_2_ (all in less than 5 min.) according to the data obtained from previous observations under the same growth conditions. Cultures, carried out in triplicate, were incubated at 28°C and at 150 rpm and the growth profile was monitored using a spectrophotometer (Eppendorf).

### RNA preparation, cDNA synthesis, and labeling

Aliquots for RNA extraction were collected after 1 h (early response) in both conditions and 10 h (late response) after inoculation in the absence of ethanol, and 28 and 40 h for the stressed cultures corresponding to the late response of the CECT10094 and Temohaya-MI26 strains, respectively. Both ethanol-treated and non-treated cells were recovered by centrifugation at 845 g, washed with diethyl pirocarbonate-treated water (Fluka, Taufkirchen, Germany). Pellets were immediately frozen in liquid nitrogen and stored at −80°C until RNA extraction. RNA extraction was carried out according to (Combina et al., 2012). RNA total concentration and purity was quantified using NanoDrop™ 1000 (NanoDrop Technologies, Wilmington, DE). The quality of the total RNA samples also was checked with an agarose gel. Fluorescently labeled complementary DNA (cDNA) was performed with “SuperScript™ Indirect cDNA Labeling System” (Invitrogen™, San Diego, CA, USA). Briefly, 20 μg of total RNA and 2.5 μg oligo (dT)_20_ (Invitrogen) were mixed in 16 μl of DEPC water. The solution was heated to 70°C for 5 min and then chilled on ice. Reverse transcription was carried out with SuperScript™ III Reverse Transcriptase, 334 μM aa-dUTP, 6 μM oligo (dT), 500 μM dATP, dCTP, dGTP, 166 μM dTTP, and 10 μM DTT in a final volume of 13 μl. The reaction was incubated at 50°C for 12 h and then with 10 μL of 1 M NaOH and 10 μL of 0.5 M EDTA for 15 min at 70°C to degrade the original RNA. pH were neutralized with 250 mM HCl. Purification of aminoallyl-cDNA (aa-cDNA) was performed with the Mini Elute PCR Purification Kit (Quiagen) according to the manufacturer's instructions. Dye coupling was performed for 2 h at room temperature and in the darkness with CyDye NHS esters (Amersham Biosciences) and 0.13 M Na_2_CO_3_ to form covalent bonds with the aa-cDNA groups. All the samples were labeled with Cy5 dye, while a reference pool was labeled with Cy3. The reference pool, which allowed the comparison of the expression differences between any samples (Gasch et al., 2000), was prepared by pooling the RNA extracted in all the samples. The amount of RNA for each sample in the pool was adjusted until approximately equal and a pool was obtained with essentially equimolar amounts of each sample. Purified labeled cDNA was then tested to verify the dye incorporation efficiency and quality using NanoDrop™ 1000. Only those samples above 100 pmol were used in the assay. No dye-swapping was carried out as the proportion of genes that had a bias in Cy3 or Cy5 incorporation was around 0.1%, and this problem is more common when cDNA labeling is performed by the direct method (Causton et al., 2003).

### Microarrays hybridization, scanning, and data analysis

The pre-hybridization step was performed in 3X SSC solution (Sigma), 0.1% SDS (Sigma) and 0.1 mg/ml BSA for 60 min at 50°C to minimize background noise and to remove friable DNA probes. Competitive hybridization was carried out manually using an equal quantity of the two labeled samples (100–200 pmol) concentrated in a Concentrator Plus (Eppendorf™, Hamburg, Germany). The mixture was resuspended in hybridization solution (5x SSC, 0.1% SDS, 50% formamide and 0.1 mg/mL salmon sperm DNA), dropped onto the Yeast 6.4 K Array (Microarray Centre, UHN, Toronto, Ontario, Canada) and covered with coverslips HybriSlip (Grace Bio-Labs, Sigma). Hybridization was conducted for 16 h in AHC chambers (Arraylt Corporation, CA, USA) and immersed in a bath at 42°C, and the labeled microarrays were washed manually with different solutions containing different SSC and SDS concentrations (Sol.1: 2x SSC, 0.1% SDS for 5 min at 42°C; Sol 2: 0.1x SSC, 0.1% SDS for 5 min at room temperature; Sol 3: 0.1x SSC for 5 min at room temperature; Sol 4: 0.01% SSC for 10 s), dried by centrifugation at 135 g for 10 min and stored in the dark. Each microarray came from a biological replicate and 24 slides were obtained.

The signal intensities of Cy3 and Cy5 were acquired with an Axon GenePix 4100 scanner (Axon Instruments, Foster city, CA, USA). Flawed or poor quality spots were manually removed from the data set and a global background subtraction was done by GenePix Pro 6.0 (Molecular Devices Corp., Union City; CA, USA). The expression ratio values (Cy5/Cy3) were transformed into logarithm base 2 to treat up- and down-regulated genes equivalently. Raw microarray data were analyzed by Acuity 4.0 (Molecular Devices Corp.), the fluorescence intensity corresponding to the two dyes was ratio-based normalized and quality control conditions were applied to remove unreliable data from the analysis. Replicates were combined and their medians were calculated, and only the spots with at least two replicates were considered. To identify the genes whose variation was due to the effect of ethanol, an indirect comparison between the slides corresponding to stressed and unstressed samples was made. The genes differentially expressed at the 95% confidence level were identified as being log_2_ (ratio) values with more than 1.96 standard deviations from the mean (Causton et al., 2003). A 5% False Discovery Rate was applied to correct for any false positives appearing. Microarray data was validated by qPCR (Supplementary Figure 1).

The enrichment of the functional categories among the up-regulated genes was analyzed using the web tool GO-TermFinder (http://go.princeton.edu/cgi-bin/GOTermFinder) and by employing Bonferroni's correction and a *p*-value threshold < 0.05. For the purpose of summarizing and removing redundant GO terms, the web server was used REViGO (Supek et al., 2011), which uses a simple clustering algorithm that relies on semantic similarity measures.

### Growth analysis under unfolded protein stress in the CECT10094 and Temohaya-MI26 strains

Inocula were prepared by introducing a single colony from the pure cultures of each strain into 5 mL of GPY. After overnight incubation at 28°C, 1 mL of each tube was centrifuged at 845 g for 5 min, and pellets were washed with sterile saline solution (0.9% NaCl), centrifuged and diluted to an optical density (OD_600_) of 0.15–0.2 in 250 μl of GPY medium modified with different concentrations (0–45 mM) of β-mercaptoethanol (Sigma-Aldrich). Growth was monitored in 96-well plates at 600 nm for 72 h in a SPECTROstar Omega instrument (BMG Labtech, Offenburg, Germany); measurements were taken every 30 min after pre-shaking for 40 s. All the experiments were carried out in triplicate under aerobic conditions and uninoculated wells for each experimental series were also included to subtract the noise signal. In this way, 114 growth curves were obtained and analyzed. In order to obtain a quantitative methodology that allows an objective and reliable comparison among yeasts, a modified Gompertz equation for decay was used to objectively estimate the NIC and the MIC for the experiments with BME (Lambert and Pearson, 2000). These parameters are related to the susceptibility and resistance of yeast to this compound, respectively. To check for significant differences for the NIC and MIC parameters, an unpaired *t*-test was performed using GraphPad Prism, version 5.0, for Windows (GraphPad Software, San Diego California USA).

### Correlation analysis between ethanol tolerance and other stresses

We analyzed the growth of 15 *S. cerevisiae* strains of different origins (Table 1) in the GPY medium modified with different stressors: 1 μg/mL tunycamicine (Sigma-Aldrich), 10% (v/v) ethanol (Scharlau Chemie S.A., Spain), 30 mM BME (Sigma-Aldrich), 3 mM oxygen peroxide (Merck Millipore), and GPY medium at pH 10. Growth was monitored with a SPECTROstar Omega instrument (BMG Labtech, Offenburg, Germany). In order to correlate ethanol tolerance with other kinds of tolerance (unfolded protein tolerance, oxidative stress tolerance, etc.), we determined the area under the OD_600_-time curve as a measure of overall yeast growth in all the strains and under all the conditions. The areas under the OD_600_-time curves were calculated by integration using the OriginPro 7.5 software (OriginLab Corporation, Northampton, USA). The relative amount of growth for each stressor was obtained by following this formula:

$$fa=({\text{area}}_{\text{test}})/({\text{area}}_{\text{cont}})$$

where *fa* is denoted as the fractional area; area_test_ is the test area (stressed) and area_cont_ is the positive control area (unstressed). We performed a correlation analysis from the plots of the *fa* (ethanol) *vs. fa* (each stressor) using GraphPad Prism, version 5.0, for Windows (GraphPad Software, San Diego California USA).

### UPR reporter assays

To measure UPR activity under ethanol stress we used a modified plasmid reporter called UPR-mCherry that encodes red fluorescent protein mCherry (Merksamer et al., 2008), driven by a minimal CYC1 promoter and four tandem unfolded protein response elements. Cells containing the plasmid were grown overnight in GPY with geneticine medium at 28°C and were allowed to reach the early exponential phase (an approximate OD600 value of 0.4) for the analysis. Then the culture was divided into sterile centrifuge tubes, pelleted and incubated with GPY media with geneticine, with or without 10% (v/v) ethanol. Cells were grown at 28°C, sampled every 2 h, pelleted and frozen in liquid nitrogen until use. GFP fluorescence was measured by flow cytometry in a LSR Fortessa flow cytometer (BD Biosciences) and analyzed with the FACS DIVA software to compile.fcs files. Files were analyzed using FloJo (Tree Star Ashland, OR). Median fluorescence intensities (MFI) were calculated for each channel and were normalized with time cero sample data. To quantify UPR induction, fractional area (fa) of 10% ethanol condition was normalized against control (see above). Biological triplicates were performed in all cases.

### Availability of supporting data

The original data from this study are available from GEO (http://www.ncbi.nlm.nih.gov/geo/) with accession number GSE44863.

## Results

### Growth of ethanol tolerant and sensitive *S. cerevisiae* strains

**Figure 1:**
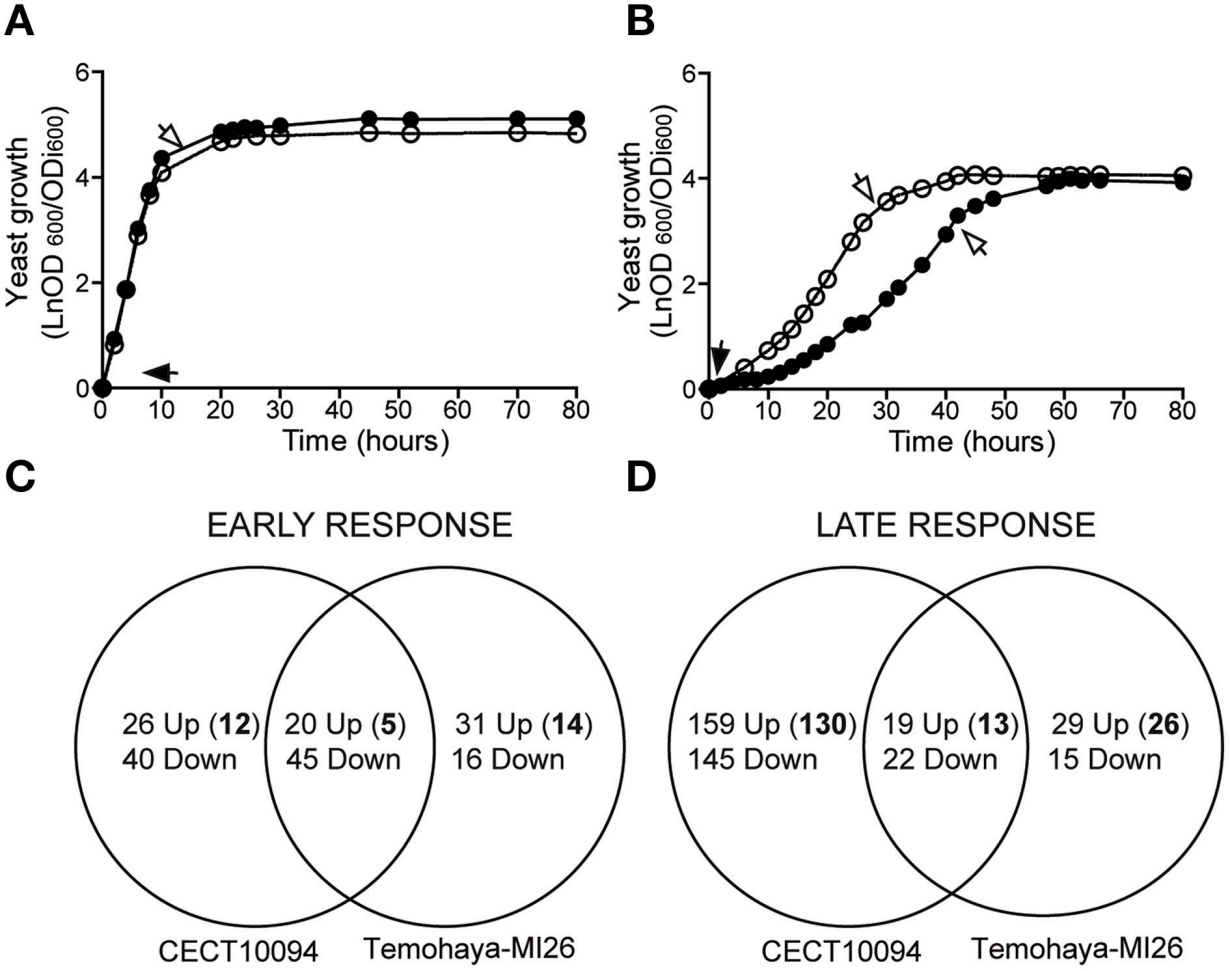
**Growth of ***S. cerevisiae*** CECT10094 (°) and Temohaya-MI26 (∙) on liquid GPY medium containing 0% (A) or 10% (B) ethanol**. Overnight yeast cells were transferred to 250 mL flasks containing 100 mL of GPY, modified or not with 10% ethanol (v/v), and initial cell density was adjusted to OD_600_ = 0.1. Arrows indicate the sampling time for the transcriptome study in the early (black arrows) and late (white arrows) response phase. Cultures were performed in triplicate and incubated at 28°C at 150 rpm. **(C,D)** Venn diagram showing the number of genes up- and down-regulated in the CECT10094 and Temohaya-MI26 strains in the early **(C)** and late response **(D)** after 10% (v/v) of ethanol addition. Bold numbers indicate the up-regulated genes not seen in previous works. For more details refer to Supplementary Table 2.

In order to elucidate transcriptional differences under ethanol stress in yeast isolates, we focused on two *S. cerevisiae* strains, CECT10094, and Temohaya-MI26. These strains were selected because they displayed very different ethanol susceptibility and resistance in minimal media (29). We evaluated the growth of these strains in GPY medium (Figure 1A) and GPY with 10% (v/v) ethanol (Figure 1B). Our results showed that ethanol resulted in a lower growth rate and an increased lag phase in both strains. Furthermore, we observed a greater growth inhibition in Temohaya-MI26 than CECT10094. The ethanol tolerant strain CECT10094 reached the exponential phase 18 h before Temohaya-MI26, and obtained a maximum specific growth rate (h^−1^) of 0.50, unlike Temohaya-MI26 whose maximum specific growth rate was 0.34. The lag period increased from 1 h at 0% ethanol to around 14 and 32 h at 10% ethanol for the CECT10094 and Temohaya-MI26 strains, respectively. In contrast, it is interesting to note that in the absence of ethanol, the Temohaya-MI26 strain reaches a 24% higher (*p* < 0.05) final population than the ethanol-tolerant strain. Therefore in view of the results, the selection of these strains was suitable to study transcriptional differences under ethanol stress in early and late growth stages.

### Global gene expression analysis in response to ethanol stress

To measure global changes in the gene expression levels of tolerant and sensible strains in early and late ethanol stress stages, the CECT10094 and Temohaya-MI26 strains were grown at 0 and 10% (v/v) ethanol in GPY medium. Gene expression variation in 10% (v/v) ethanol respect to 0% of both strains was compared in the early (1 h) or late (after exponential phase) response to ethanol stress (see arrows in Figure 1). After filtering and normalization, a complete dataset of genes, considered to be significantly down- or up-regulated, was obtained (Supplementary Table 1). In the early response, 178 genes were differentially expressed in both strains (Figure 1C). A total of 46 genes were up-regulated in the tolerant strain, of which 26 were exclusively up-regulated in this strain and some were related with the cellular response to oxidative stress (*YDL124W, GRE3* and *NCE103*), transport and utilization of proline (*PUT4* and *PUT1*), synthesis of mono-unsaturated fatty acids (*OLE1*), molecular chaperones like *HSP104* and *HSP32*, protein folding (*MPD1*) or energy reserve metabolic processes (*RGI1*), and have already been described as key ethanol-tolerance genes. Both strains shared 20 up-regulated genes, the majority of which were related with Heat Shock Proteins (*HSP42, HSP12, HSP26, HSP31*, and *HSP36*), acetyl-CoA biosynthesis, glycolysis, and nitrogen utilization. In the Temohaya-MI26 strain, specifically up-regulated genes (Shobayashi et al., 2007) were related with the stress response (*DDR2, ALD3*, and *GRE1*), sporulation, cell wall organization and unknown function genes. As expected, after 1 h of ethanol stress, a large number of down-regulated genes encoded components of the protein synthesis machinery. This group represented 64.3 and 70.5% of all the down-regulated genes in CECT10094 and Temohaya-MI26, respectively. All the up-regulated genes after 1 h of ethanol stress for both strains are compiled in Supplementary Table 2.

In the late response, 389 genes were differentially expressed in both strains (Figure 1D). A total of 178 genes were up-regulated in the CECT10094 strain, of which 159 were exclusively up-regulated in this strain. Some were related to protein folding (*ERO1, LHS1, SBA1*, and *SSE2*), ATP synthesis (*ATP1*-*4, ATP7*, and *ATP20*), ergosterol biosynthesis (*ERG5* and *ERG20*), heat shock protein (*HSP104, HSP31*, and *HSP82*), long-chain fatty acid transport (*FAT1*), phosphatidylcholine biosynthesis (*CHO2*), vacuolar acidification (*VMA8* and *VMA10*), Ty element transposition (*YMR051C, YJR028W, YAR009C, YJR026W, YML045W, YML040W, YMR046C, YAR010C*, and *YCL020W*), removal of superoxide radicals (*CUP1-1*), and protein degradation (*CDC34, PRE1, PRE4, PRE6*-*7, PUP2, RPN12, RPN5, RPN8, RPT4*-*5, SCL1*, and *UBC1*). The two strains shared 19 up-regulated genes, which were mainly related with the response to stress (*HSC82, YHB1*, and *MSN4*), cell wall organization and biogenesis (*YGP1* and *ECM4*) and amino acid transport (*BAP2* and *TAT1*). In the Temohaya-MI26 strain, there were 29 specifically up-regulated genes, some of which were related with the alcohol metabolic process (*ADH6*), heat shock protein (*HSP12*) and sporulation (*UBX6* and *DIT1*). All the up-regulated genes in the late response for both strains are compiled in Supplementary Table 2. It is noteworthy that in the genes expressed only in tolerant strain CECT10094 after 1 h under ethanol stress, the GO-Term analysis identified GO categories that were related to the response to oxidative stress, proline metabolic process and thiamine-containing compound biosynthesis, unlike the less tolerant strain, which did not present GO terms related with known ethanol tolerance mechanisms (Table 2). In the late response, CECT10094 showed GO terms related with the protein folding, proteosomal ubiquitin-independent catabolic process, RNA-mediated transposition, ATP synthesis coupled proton transport, and proton transport. At this time point, Temohaya-MI26 did not show GO terms relate with ethanol tolerance mechanisms either.

**Table 2:** **Enrichment of the functional categories of the up-regulated genes during ethanol stress in the CECT10094 and Temohaya-MI26 strains^a^**.

**GO ID**	**Biological process**	***p*-value**	**Up-regulated genes**	**Total genes**
**EARLY PHASE**				
**Up-regulated genes in the CECT10094 strain under ethanol stress**				
GO:0006979	Response to oxidative stress	0.0052	6	91
GO:0019321	Pentose metabolic process	0.0170	3	14
GO:0055114	Oxidation-reduction process	0.0001	14	450
GO:0044281	Small molecule metabolic process	0.0054	17	49
GO:0042724	Thiamine-containing compound biosynthetic process	0.0259	3	16
GO:0006560	Proline metabolic process	0.0393	2	9
**Up-regulated genes in the Temohaya-MI26 strain under ethanol stress**				
GO:0055114	Oxidation-reduction process	0.0001	15	450
GO:0016052	Carbohydrate catabolic process	0.0048	7	120
GO:0046365	Monosaccharide catabolic process	0.0214	5	66
GO:0006098	Pentose-phosphate shunt	0.0291	3	15
**LATE PHASE**				
**Up-regulated genes in the CECT10094 strain under ethanol stress**				
GO:0010499	Proteasomal ubiquitin-independent protein catabolic process	0.0005	6	14
GO:0032196	Transposition	0.0053	13	114
GO:0032197	Transposition, RNA-mediated	0.0190	12	110
GO:0055114	Oxidation-reduction process	0.0105	28	450
GO:0090342	Regulation of cell aging	0.0459	3	4
GO:0006122	Mitochondrial electron transport, ubiquinol to cytochrome c	0.0031	5	11
GO:0015986	ATP synthesis-coupled proton transport	0.0062	7	30
GO:0042026	Protein refolding	0.0375	5	17
GO:0022900	Electron transport chain	0.0036	10	64
GO:0015992	Proton transport	0.0293	7	42
**Up-regulated genes in the Temohaya-MI26 strain under ethanol stress**				
GO:0006177	GMP biosynthetic process	0.0007	3	5
GO:0015864	Pyrimidine nucleoside transport	0.0118	2	2
GO:0009123	Nucleoside monophosphate metabolic process	0.0418	4	42
GO:0009163	Nucleoside biosynthetic process	0.0271	5	70

a *GO categories were analyzed using the GO-TermFinder web tool (http://go.princeton.edu/cgi-bin/GOTermFinder) and employing Bonferroni's correction and a p-value threshold of 0.05, redundant GO terms were removed using the REViGO web server (http://revigo.irb.hr/)*.

It should be noted that, although more genes were up-regulated in the late response (207 genes), only 17.96% of them were associated with the ethanol stress response in previous studies with microarrays, while the up-regulation of 59.74% of our genes in the early response (77 genes) has been observed in previous studies (Supplementary Table 2), probably because most microarrays studies done to date have focused on early stages of ethanol stress response.

### Unfolded protein response and ethanol stress

Taking into account the presence of up-regulated genes associated with the accumulation of unfolded proteins and GO categories related with protein refolding in the most tolerant strain under ethanol stress, we explore the possibility that the unfolded protein response, mediated by transcription factor Hac1p, was implicated in ethanol tolerance. Based on this premise, we carried out a promoter analysis of those genes overexpressed specifically in each strain under ethanol stress to determinate the percentage of the up-regulated genes directly related with transcription factor Hac1p during ethanol stress. A search in the YEASTRACT database revealed that 19.23% of the genes exclusively overexpressed in the early phase of ethanol stress and 5.73% of the genes exclusively overexpressed in the late phase of ethanol stress presented regulatory associations with Hac1p in the most ethanol-tolerant strain CECT10094 while Hac1p regulated genes were not found in the Temohaya-MI26 strain in either growth stage (Table 3). This evidence suggests that increased tolerance to ethanol can be related to an enhanced unfolded protein response (UPR). Other stress-related transcription factors, such as Msn2p/Msn4p for general stress, Hsf1p for heat stress, and Yap1p for oxidative stress, were also found (Table 3).

**Table 3:** **A promoter region analysis showing % of the up-regulated genes containing binding sites exclusively in the CECT10094 and Temohaya-MI26 strains after addition of ethanol**.

**Transcription Factor**	**CECT10094**		**TEMOHAYA-MI26**	
	**Early (%)**	**Late (%)**	**Early (%)**	**Late (%)**
Msn2p	65.4	32.5	90.3	35.7
Msn4p	65.4	28.0	87.1	42.9
Hsf1p	61.5	17.2	48	17.9
Hac1p	19.2	5.7	0.0	0.0
Yap1p	53.9	29.3	45.2	39.3

*Promoter regions were analyzed by YEASTRACT database in order to find transcription factor-binding sites*.

**Figure 2:**
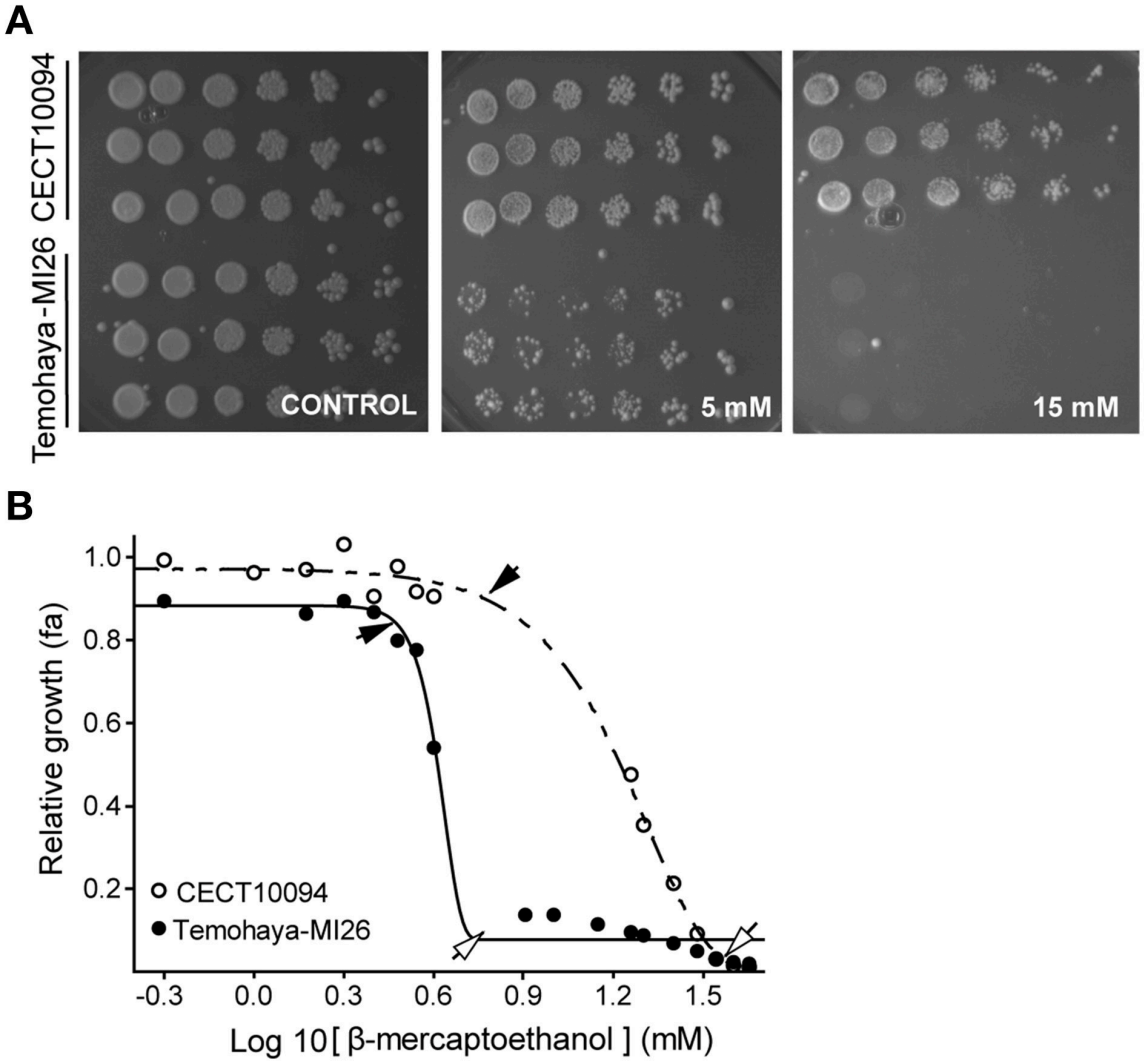
**Tolerance to the protein denaturing agent BME in CECT10094 and PE35M strains. (A)** Drop test analysis in GPY media and in presence of 5 or 15 mM BME. The plates were grown for 6 days at 28°C. **(B)** Estimation of the minimum inhibitory concentration (empty arrows) and the non-inhibitory concentration (black arrows) of the CECT10094 and Temohaya-MI26 strains to protein denaturing agent β-mercaptoethanol. Curve fitting was achieved with a modified Gompertz function for decay.

In order to correlate the Hac1p-mediated UPR response with ethanol tolerance, we studied the growth of the Temohaya-MI26 and CECT10094 strains at different concentrations of endoplasmic reticulum (ER) stressor β-mercaptoethanol (BME), which prevents disulfide-bond formation. The results showed that Temohaya-MI26 plate growth was significantly affected with the presence of 5 but especially with 15 mM of BME in GPY medium (Figure 2A). In contrast, CECT10094 growth was not significantly affected by BME. With the purpose of clarify this physiological difference among both strains we estimated the non-inhibitory concentration (NIC) and the minimum inhibitory concentration (MIC) by studying yeasts growth in flasks with a range of BME concentrations. Figure 2B shows the curve fitting for both strains with an *R*^2^ ranging from 0.96 to 0.99. The CECT10094 and Temohaya-MI26 strains gave significant differences in the NIC values of 6.54 ± 1.48 mM and 2.80 ± 0.90 mM, and in the MIC values of 36.74 ± 2.96 mM and 7.29 ± 2.04 mM, with a *p*-value of 0.01 and 0.0001 for the NIC and MIC values, respectively. Hence the Temohaya-MI26 strain gave lower NIC and MIC values than the CECT10094 strain, which is indicative of higher susceptibility to the protein denaturant BME. This experiment suggests that strains with an enhanced UPR also have increased ethanol tolerance.

To further ascertain the correlation between UPR and ethanol tolerance, we analyzed the growth of 15 *S. cerevisiae* strains in GPY medium modified with different stressors, such as tunycamicine, ethanol, BME, oxygen peroxide, and GPY medium at pH 10. Then we studied the correlation between ethanol and ER stress and compared it to other stresses, such as oxidative, osmotic, and high pH stress. The correlation analysis (Table 4) showed that tolerance to ethanol and protein folding stress (BME and tunycamicine) correlated significantly, whereas other stresses showed no correlation.

**Table 4:** **Correlation between yeast population behavior after treatment with different agents**.

	**10%**	**BME**	**H_2_O_2_**	**pH 10**	**TM (1 μg/mL)**
	**Ethanol**	**(30 mM)**	**(3 mM)**		
10% Ehanol	–	**0.02**	0.29	0.05	**0.01**
BME (30 mM)		–	0.08	0.24	**0.01**
H_2_O_2_ (3 mM)			–	0.05	0.12
pH 10				–	0.08
TM (1 μg/mL)					–

*Bold numbers indicate correlation between stressors (p < 0.05)*.

**Figure 3:**
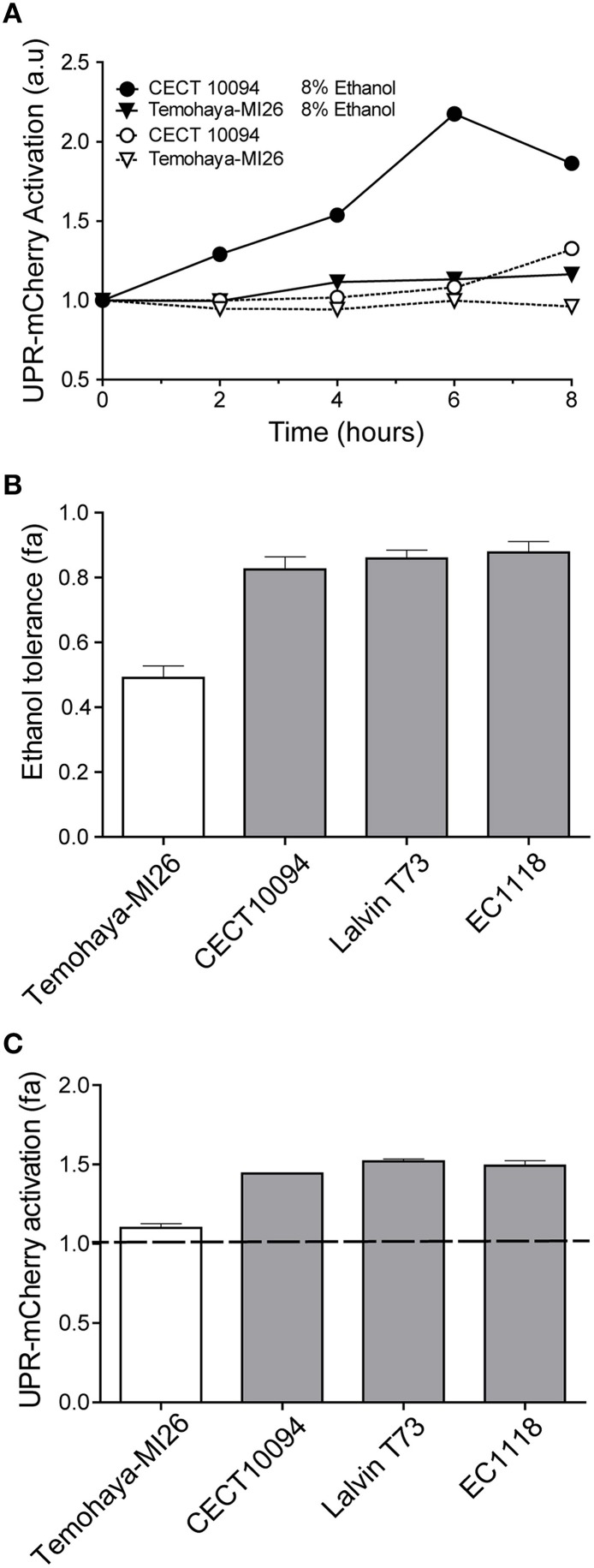
**Relation between ethanol tolerance and UPR signaling under ethanol stress**. **(A)** UPR activation was analyzed measuring the fluorescence intensity by flow cytometry in Temohaya-MI26 and CECT10094 strains containing UPR mCherry reporter after resuspension of exponentially growing cells in control medium or containing 10% of ethanol. Experiments were done in triplicate and data were normalized to its initial point. Calculated standard deviations did not exceed 5% of the average. **(B)** Ethanol tolerances in Temohaya-MI26, CECT 10094, Lalvin T73, and EC1118 strains were determined comparing yeast growth curve in GPY and GPY modified with 10% ethanol. Fractional area (fa) was calculated normalizing the area under the curve in GPY 10% ethanol respect to control. Three biological replicates were done and the mean value and standard deviation is shown. **(C)** UPR-mCherry activation in response to ethanol stress in Temohaya-MI26, CECT 10094, Lalvin T73, and EC1118 strains. The fractional area of mCherry intensity was obtained by the quotient between the area under the curve of the intensity of the reporter in GPY medium with ethanol (10%) and control medium after 6 h of growth. The dotted line shows the threshold value above which the activation of the reporter is higher than in non-stress conditions (control). Gray bars represent the strains isolated from wine fermentation while the white bar shows the strain isolated from traditional fermentations.

After confirm the physiological relation among ethanol stress and unfolded protein response we wanted to determine if ethanol stress induced UPR activation. To test this hypothesis we used a previously described mCherry UPR reporter containing four Hac1p binding sites. After marker swap, we introduced the plasmid with the reporter in the Temohaya-MI26 and CECT10094 strains. Finally, modified strains were inoculated in complete media with or without 10% ethanol and samples were taken to observe UPR activation by flow cytometry. The results (Figure 3A) show that any strain increased mCherry levels in complete media without ethanol during the first 6 h. Also, almost no activation was observed in Temohaya-MI26 strain with 10% ethanol in the media. However, the ethanol tolerant strain CECT10094 increased mCherry levels when 10% ethanol was present in the media, reaching a maximal value of 2.2-fold after 6 h. This result confirmed that ethanol stress activates UPR, especially in ethanol tolerant strains.

The different levels of UPR activation after ethanol stress in Temohaya-MI26 and CECT10094 prompted us to investigate whether other ethanol tolerant strains have an increased UPR after ethanol stress. To evaluate this hypothesis we selected two different industrial *S. cerevisiae* wine strains previously characterized as ethanol tolerant strains, T73 and EC1118. First we evaluated ethanol tolerance by following cell growth after 10% ethanol stress (Figure 3B). The results show that wine strains show high levels of ethanol tolerance as CECT10094, comparing to Temohaya-MI26. Next we wanted to evaluate UPR activation in response to ethanol in the same strains and for that we performed the same experimental set up as described previously for CECT10094 and Temohaya-MI26 (Figure 3C). The UPR mCherry reporter activation for all the strains was quantified and represented in Figure 3C. The results showed that strains with high ethanol tolerance also present UPR activation whereas Temohaya-MI26, showing low ethanol tolerance, showed almost no UPR activation. These results also point to a functional role of the UPR in the ethanol tolerance in yeast cells and suggest substantial degree of phenotypical diversity among the different *S. cerevisiae* strain types regarding UPR after cytotoxic ethanol effects.

## Discussion

### Early and late response to ethanol stress

In the present work, we analyzed yeast responses against ethanol stress in two strains which differed in terms of ethanol tolerance in two growth stages. Our results revealed that optimal tolerance of yeasts to ethanol implied a series of dynamic events prolonged in the time rather than a short transient response. Our results suggest that ethanol stress-essential genes may be more relevant for long-term adaptation or for subsequent stresses than the immediate response to stress, and that the network of induced genes is more important for adaptation to stress than the effect of a single gene. Besides we observed very dynamic responses (only 3.5 and 3.22% of commonality comparing both time points analyzed in the CECT10094 and Temohaya-MI26 strains, respectively) which hints that ethanol yeast tolerance is a procedure involving numerous events that interact over time.

According to the transcriptomic analysis, in the early ethanol stress stages, the ethanol-tolerant CECT10094 strain showed an overexpression of the genes related to transport and utilization of proline, synthesis of mono-unsaturated fatty acids, molecular chaperones, and response to oxidative stress, which were not seen in the less tolerant strain. Some of these genes have been previously described as key ethanol-tolerance genes that counteract the fluidizing effect of ethanol, inhibit aggregation during protein refolding and reduce the reactive oxidative species (ROS) produced during ethanol stress (Alexandre et al., 2001; Chandler et al., 2004). Several genes involved in NADH/NADPH regeneration were up-regulated in the CECT10094 strain in early stages of ethanol response, including *ZWF1, SOL4*, and *ALD4*. NADPH is required by glutathione and thioredoxin as a reducing agent to reduce oxidized glutathione (GSSG) and thioredoxin, these being key elements to counteract oxidative stress. In addition, NADPH is necessary to carry out NADH-dependent desaturation of stearic acid in oleic acid, and is considered the main determinant of ethanol tolerance in *S. cerevisiae* (You et al., 2003), formed by the catabolic membrane desaturase encoded by *OLE1*, which is also up-regulated in the CECT10094 strain. In contrast to other studies (Chandler et al., 2004; Ma and Liu, 2010a), our data and Shobayashi et al. (2007) have observed enhanced mRNA levels of *OLE1* in the early stages of ethanol stress. This suggests that not only mRNA stability, translation, and enzyme reactions with carbon and oxygen sources regulate unsaturated fatty acid (UFA) biosynthesis (Martin et al., 2007), but transcription levels can also play a more important role than previously thought.

In the late ethanol stress response, we found that the genes related with energy generation (*ATP1, ATP3, ATP4, ATP7*, and *ATP20*) were significantly overexpressed in the most tolerant strain. This fact indicates that under ethanol stress conditions, increased ATP synthesis might be responsible for the activation of protein synthesis and proton transportation through the plasma membrane (Rosa and Sá-Correia, 1991). This is consistent with the overexpression of *VMA* genes (*VMA10* and *VMA8*) that encode vacuolar H^+^-ATPase (V-ATPase), an electrogenic proton pump involved in vacuolar acidification and, therefore, in the compensation of H^+^ entry induced by ethanol (Fujita et al., 2006). Higher ergosterol content in yeast has been associated with greater ethanol tolerance by preventing interdigitation and maintaining optimal membrane thickness (Vanegas et al., 2012). We noticed the overexpression of the genes related to the ergosterol pathway (*ERG5, ERG11*, and *ERG20*), which has not been previously observed in microarrays studies (Alexandre et al., 2001; Chandler et al., 2004; Teixeira et al., 2009; Ma and Liu, 2010a). It is noteworthy that the genes related with actin cytoskeleton organization, such as *RDI1, RHO1, ARC19*, and *ARP2*, were overexpressed in CECT10094. According to Kubota et al. (2004), the spatial organization of the F-actin cytoskeleton is transiently disrupted by the addition of ethanol. Thus, the CECT10094 strain can be expected to counteract the depolarization of F-actin more efficiently than the less tolerant strain to obtain a higher growth rate under stressful conditions.

Our results revealed that the retrotransposon-related Gene Ontology (GO) categories were highly up-regulated in CECT10094 strain, while the Temohaya-MI26 strain did not overexpress any retrotransposon-related gene. Little attention has been paid to retrotransposon-related gene activation after ethanol stress, probably due to the misinterpretation of transcriptomes and databases (Stanley et al., 2010b). Although the adaptive significance of this activation remains unclear, we observed overexpressed levels of *GCN4*, whose protein Gcn4p has been suggested to be a Ty1 activator in stress (Morillon et al., 2002). Further work is required to determine whether an association exists between retrotransposons and the ethanol stress response in yeast.

### Ethanol stress triggers UPR activation

One very interesting finding among our results is the observation that the ethanol-tolerant CECT10094 strain overexpressed genes whose GO was related to protein catabolism and refolding in the late ethanol stress phase, mainly regulated by Hac1p, a UPR-specific transcription factor that induces UPR target gene expression, including ER resident chaperones and critical protein-folding enzymes to restore ER protein-folding homeostasis (Travers et al., 2000; Ron and Walter, 2007; Kimata and Kohno, 2011). Indeed, we observed more genes with Hac1p binding sites, in the most tolerant strain in both growth stages, as well as UPR target genes, such as *LHS1, ERO1*, and *KAR2*, which encoded redox proteins and ER chaperones and were up-regulated in the CECT10094 strain. The correlation between ethanol and unfolded protein tolerance (Table 4) suggests that strains with an enhanced unfolded protein response also increase ethanol resistance. Furthermore, our results confirmed that ethanol triggers the UPR response in the cell and that the conjunction between UPR response and ethanol tolerance may be an interesting way to explain how yeast overcomes this stress. To date, ethanol adaptation has been related with the coordinate action of transcription factors Msn2/4p, Yap1p, and Hsf1p to control general stress, oxidative stress and heat shock responses, respectively, in yeast (Ma and Liu, 2010a,b).

Ethanol and UPR signaling activation has been previously correlated in human cells (Pandol et al., 2010; Ji, 2012). The unfolded protein response (UPR) is a complex pathway triggered by ER stress to enhance and restore the protein-folding and secretory capacity of the ER, hence its activation under ethanol stress is conserved across vertebrates. Likewise, alcohol damages mammalian cells and induces numerous pathological stress responses, such as the ER stress response, which has recently emerged as a novel mechanism for pancreas and liver disease in chronic alcoholism. In fact, a recent study has correlated pancreas disease with an insufficient UPR response, and ER stress-induced overproduction of lipids can lead to fatty liver in alcoholic patients (Ji and Kaplowitz, 2003; Pandol et al., 2010). These data support our findings and suggest that UPR activation after UPR stress can be conserved in eukaryotes.

Interestingly, not all strains activate UPR at the same level after ethanol stress. This suggests that the strains that have been adapting their physiology to elevated amounts of ethanol, as it is found in fermentative process as winemaking, rely on UPR signaling to resist this suboptimal condition. All this data pointed out that there are possibilities to improve the ethanol tolerance of selected yeast starters via the enhancement of UPR. Also, the important differences that can be observed among the different strains suggest that UPR pathway can be a key modulator to adapt yeast cells to the different environments.

In summary, this work shed light on the ethanol transcriptional stress response, demonstrates the role of the UPR pathway under ethanol stress and links this response to an enhanced ethanol tolerance. These data uncover potential applications to increase ethanol tolerance of yeasts. Many industrial applications, such as winemaking, depend on yeast tolerance to ethanol. Thus our research opens new line of possibilities related to UPR pathway enhancement to increase ethanol stress resistance. Either selecting natural or engineered strains with elevated UPR response will be a new field for industrial yeast strain improvement. Future experiments will shed more light on the specific signal that triggers UPR activation during ethanol stress.

## Author contributions

AQ, EN, and RP conceived and designed the experiments. EN and RK performed the experiments. AQ, EN, RP, and RK analyzed the data. EN contributed reagents/materials/analysis tools. AQ, EN, and RP wrote the paper.

### Conflict of interest statement

The authors declare that the research was conducted in the absence of any commercial or financial relationships that could be construed as a potential conflict of interest.

## Supplementary material

Supplementary Figure1 **Microarray data validation by qPCR (eps file,.eps)**. mRNA of significantly activated genes *ACS1, OPI3, TFS1* (in Temohaya-MI26 and CECT10094) and *ERO1, ALD4* y *HSC82* (in CECT10094) was measured by RT-qPCR, using *ACT1* and 18S ribosomal gene as reference. High relation between microarray and RT-qPCR data was observed, showing a Spearman correlation coefficient (ρ) of 0.833 (*p* = 0.008; **A**). Also, Bland-Altman analysis to study method concordance showed a high similar outcome of both techniques (95% confidence interval; **B**). The x-axis represents the average fold change of each sample measured by quantitative reverse transcription PCR (RT-qPCR) and microarrays. The y-axis is the difference in fold change calculated by microarray measurements minus the RT-qPCR measurements for each sample. The solid line (*y* = −0.52) is the mean difference in fold change of all the samples. The two dotted lines represent 1.96 standard deviations from the mean difference.

Supplementary Table1 **Gene expression under ethanol stress (excel file,.xlsx)**. Comparison of the mRNA expression of *S. cerevisiae* CECT10094 and Temohaya-MI26 by fold changes after 1 h and after exponential phase after addition of ethanol to the GPY medium. The expressions for each gene at each time point were represented in the relative fold changes against themselves under non-stressed conditions. Differentially expressed genes were identified as log_2_ (ratio) values of the more than 1.96 standard deviations from the mean (at the 95% confidence level). Red indicates overexpression, green is repressed expression, and yellow implies no significant changes.

Supplementary Table2 **Up-regulated genes in ***S. cerevisiae*** CECT 10094 and Temohaya-MI26 after 1 h and after exponential phase of ethanol stress (excel file,.xlsx)**. Promoter regions were analyzed using Regulatory Sequence Analysis Tools (RSAT) (http://rsat.ulb.ac.be/rsat). The number of binding sites for transcription factors involved in various stress responses, such as Msn2p/Msn4p for general stress, Hac1p for unfolded protein response, Hsf1p for heat stress, and Yap1p for oxidative stress, are shown. The number of STRE elements in their promoter regions, implicated in a general stress response in yeast, was also analyzed. Those genes previously found to be up-regulated under ethanol stress are underlined.
