# Supplementary figures and images for: Ethanol Cellular Defense Induce Unfolded Protein Response in Yeast
Source: Front Microbiol. 2016 Feb 18;7:189. doi: 10.3389/fmicb.2016.00189 (PMC4757686; doi:10.3389/fmicb.2016.00189)

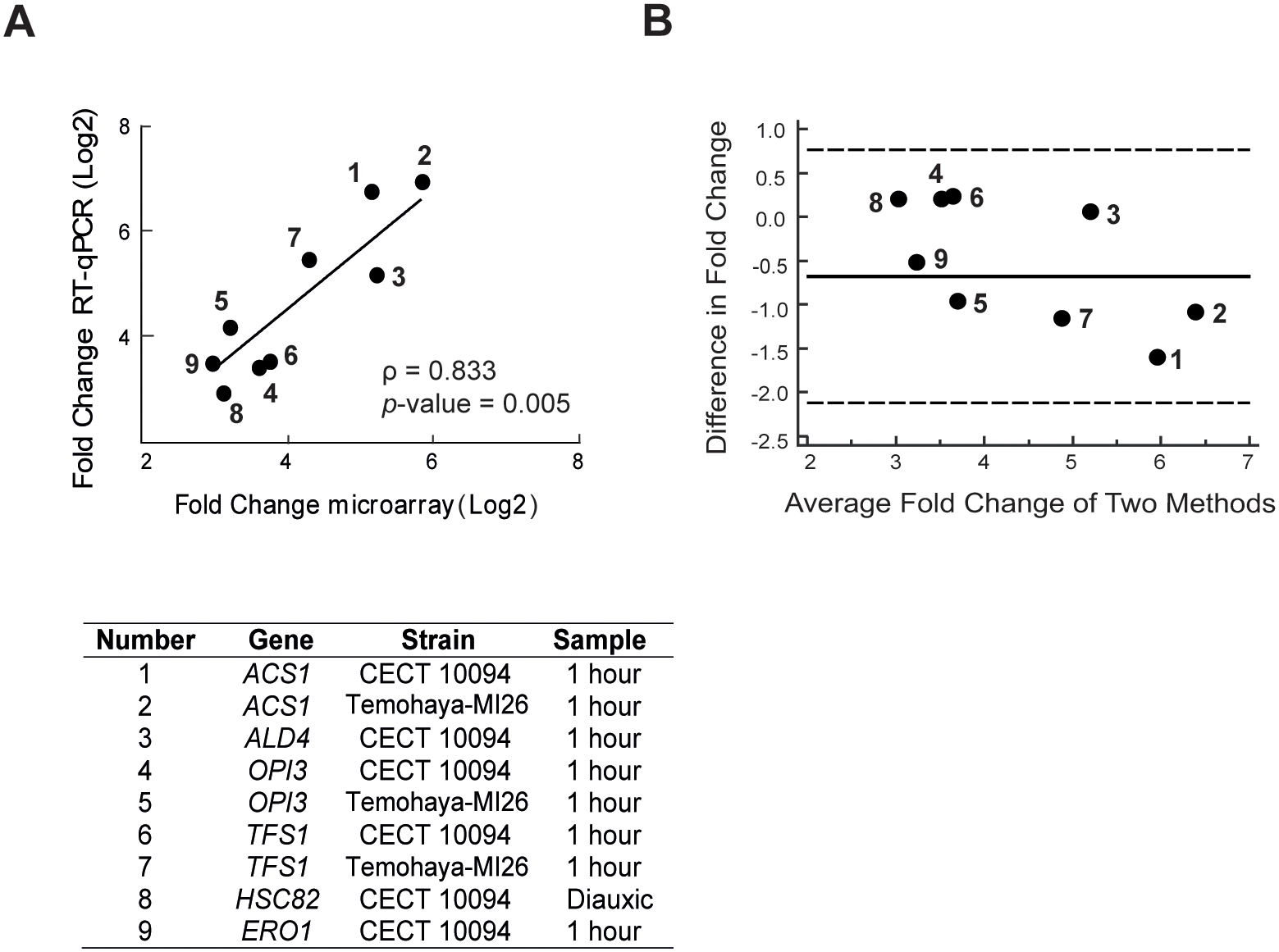

Supplement: Supplementary Figure1 — Microarray data validation by qPCR (eps file,.eps). mRNA of significantly activated genes ACS1, OPI3, TFS1 (in Temohaya-MI26 and CECT10094) and ERO1, ALD4 y HSC82 (in CECT10094) was measured by RT-qPCR, using ACT1 and 18S ribosomal gene as reference. High relation between microarray and RT-qPCR data was observed, showing a Spearman correlation coefficient (ρ) of 0.833 (p = 0.008; A). Also, Bland-Altman analysis to study method concordance showed a high similar outcome of both techniques (95% confidence interval; B). The x-axis represents the average fold change of each sample measured by quantitative reverse transcription PCR (RT-qPCR) and microarrays. The y-axis is the difference in fold change calculated by microarray measurements minus the RT-qPCR measurements for each sample. The solid line (y = −0.52) is the mean difference in fold change of all the samples. The two dotted lines represent 1.96 standard deviations from the mean difference. [file Image1.TIF]
